# On the Dropsy of Pregnancy

**Published:** 1856-08

**Authors:** 


*On the Dropsy of Pregnancy.* By M. Becquerel.

Four forms of dropsy are observed in pregnant women, which are far from being of the same importance.

1. *Mechanical Dropsies,* perhaps the most common, are due to the pressure exerted by the gravid uterus, their production being favored by the lesser density of the blood in pregnant women, and the slight diminution of albumen that exists in its serum. These dropsies are confined to the lower extremities, are of no importance beyond their inconvenience, and disappear after delivery.

2. *Dropsies due to Changes in the Blood, but unaccompanied by Albuminuria.*—The change in the blood which induces these dropsies, consists in a diminution in the amount of the albumen of the serum, a diminution that is sometimes considerable, and for which we can assign no other cause than the fact of the pregnancy, and its influence on the various immediate principles of the blood. This description of dropsy, like the two next, tends to become general. It is of importance to distinguish it from the two others, and especially the 4th, for it does not predispose to eclampsia. It is by analysis of the blood alone that we can establish its existence. It disappears also after pregnancy, but far more slowly. It has been observed that women suffering from it remain feeble for a long period, their "getting up" being slow and difficult.

3. *Dropsies with Changes in the Blood and Albuminuria, but without Bright's Disease, properly so called.*—These dropsies are the consequence of the diminution of the albumen of the blood, produced by its deperdition through the kidney. Until lately it was supposed that such loss might take place without material lesion of the kidney; but from the investigations made by M. Robin and the author, it results that this albuminuria is due to a special modification taking place in the epithelial cells of the tubuli, a modification consisting in the infiltration of the cells and tubuli by numerous granules of a proteric nature. This infiltration is analogous to that which M. Robin had already found in choleraic albuminuria, and like it is susceptible of cure. The absolute diagnosis during life of this disease from Bright's affection is very difficult, and yet it is highly important, as the prognosis must be entirely based upon it. It is in women who are the subjects of these dropsies that we have to fear eclampsia, and the predisposition to puerperal peritonitis. Eclampsia is not, however, a necessary consequence; and when we find general dropsy, change in the blood, and albuminuria co-existing, we still cannot affirm that this terrible accident will follow. On the other hand, whenever we find eclampsia we are certain of finding, not only dropsy, but albuminous urine, and change in the blood. In respect to the termination of this form of dropsy it may be observed, that if eclampsia does not supervene, a cure is almost certain, while, in the case of its occurring the result is dependent upon that of the eclampsia.

4. *Dropsies due to Bright's Disease.*—It is very important to establish the diagnosis of this form. We may lay stress upon the somewhat larger quantity of albumen, the presence of fragments of the tubuli, of fibrinous filaments, and fatty globules. When eclampsia complicates this form it is invariably fatal; and even when eclampsia does not occur, the disease is not arrested after delivery. The dropsy continues to increase, the termination proving, after a certain period, fatal.—*London Med. Times, from Rev. Medico-Chirurgicale.*

